# Efficacy, safety, and long-term survival of concomitant valve replacement and bipolar radiofrequency ablation in patients aged 70 years and older: a comparative study with propensity score matching from a single-Centre

**DOI:** 10.1186/s13019-020-01322-9

**Published:** 2020-10-02

**Authors:** Zhi-qin Lin, Zeng-rong Luo, Qian-zhen Li, Liang-wan Chen, Feng Lin

**Affiliations:** Department of Cardiovascular Surgery, Union Hospital, Fujian Medical University, Xinquan Road 29#, Fuzhou, Fujian 350001 People’s Republic of China

**Keywords:** Elderly patients, Atrial fibrillation, Bipolar radiofrequency ablation, Propensity-score matching

## Abstract

**Background:**

Concomitant bipolar radiofrequency ablation and valve replacement in the elderly remains controversial. In the current study, we aimed to compare the outcomes of concomitant valve replacement and bipolar radiofrequency ablation with valve replacement alone in elderly patients with atrial fibrillation (AF).

**Methods:**

This was a retrospective study of patients aged ≥70 years who underwent valve replacement with or without bipolar radiofrequency ablation in a single-centre between January 2006 and March 2015. The early postoperative results and long-term clinical outcomes were compared after propensity score matching.

**Results:**

A total of 34 pairs of patients (73.94 ± 2.64 years old; 34 in the AF with ablation group and 34 in the AF without ablation group) were enrolled in the propensity score matching analysis. There were no significant differences between the two matched groups in terms of surgical mortality (5.88% vs. 2.94%, *P* = 0.555) and major postoperative morbidity. Kaplan–Meier analysis revealed a significantly better overall survival in the AF with ablation group compared to the AF without ablation group (*P* = 0.009). Cumulative incidence curves showed a lower incidence of cardiovascular death in the AF with ablation group (*P* = 0.025, Gray’s test). Patients in the AF with ablation group had a reduced incidence of stroke compared to patients in the AF with ablation group (*P* = 0.009, Gray’s test). The freedom from AF after 5 years was 58.0% in the AF with ablation group and 3.0% in the AF without ablation group.

**Conclusions:**

The addition of bipolar radiofrequency ablation is a safe and feasible procedure, even in patients aged ≥70 years, with a better long-term survival and a reduced incidence of stroke compared to valve replacement alone. These findings suggest that bipolar radiofrequency ablation should always be considered as a concomitant procedure for elderly patients with AF who require cardiac surgery. However, a large-scale, prospective, multi-centre, randomized study should be performed in the future to fully validate our findings.

## Background

Atrial fibrillation (AF) is a common cardiac arrhythmia among older adults [1], which is known to increase the risk of myocardial infarction, heart failure, stroke, dementia, and mortality [2]. Caswell et al. [3] demonstrated that postoperative AF was a risk factor of cardioembolic stroke after valve replacement, and restoration of sinus rhythm is considered to be beneficial for improving long-term outcomes [4]. Surgery for AF has been shown to be feasible and effective, and does not increase perioperative morbidity and mortality when combined with other cardiac procedures [5–7]. While effective, the Cox-maze III procedure has limitations for the treatment of AF, as it is both invasive and time consuming. Recent techniques, including bipolar radiofrequency ablation (BRFA), have emerged as alternative treatment options for AF.

Patients with advanced age may have an increased frequency of adverse side effects to drugs used for treating AF [8]. In addition, age is deemed to be an independent predictor of stroke in patients with AF [9]. Age of≥70 years is frequently accompanied by multiple co-morbidities, and is a significant risk factor associated with worse postoperative results [10–13]. Thus, whether concomitant BRFA should be performed in elderly patients is controversial. Several previous studies have indicated similar success rates and similar low complication rates in the elderly compared to younger patients undergoing concomitant Cox-maze surgery [14, 15]. In an analysis from the Polish National Registry of Cardiac Surgery Procedures [14], neither clinical benefit nor harm was demonstrated in a subgroup of patients > 70 years with concomitant surgical ablation during the 12-year study period. Furthermore, Funatsu et al. [16] set inclusion criteria for adding surgical ablation, including an age of < 70 years in their clinical practice. However, patients with severe conditions may prefer to choose a simplified procedure without surgical ablation. Furthermore, no previous clinical studies have specifically compared the clinical results of concomitant surgical ablation for AF vs. valve replacement (VR) alone in the elderly, and few studies have assessed the effect of surgical ablation on long-term outcomes [17]. Therefore, in the current study, we aimed to compare the clinical results between concomitant BRFA and no ablation in patients with VR aged ≥70 years after adjusting for severity by propensity score matching (PSM).

## Patients and methods

### Study populations

**Fig. 1:**
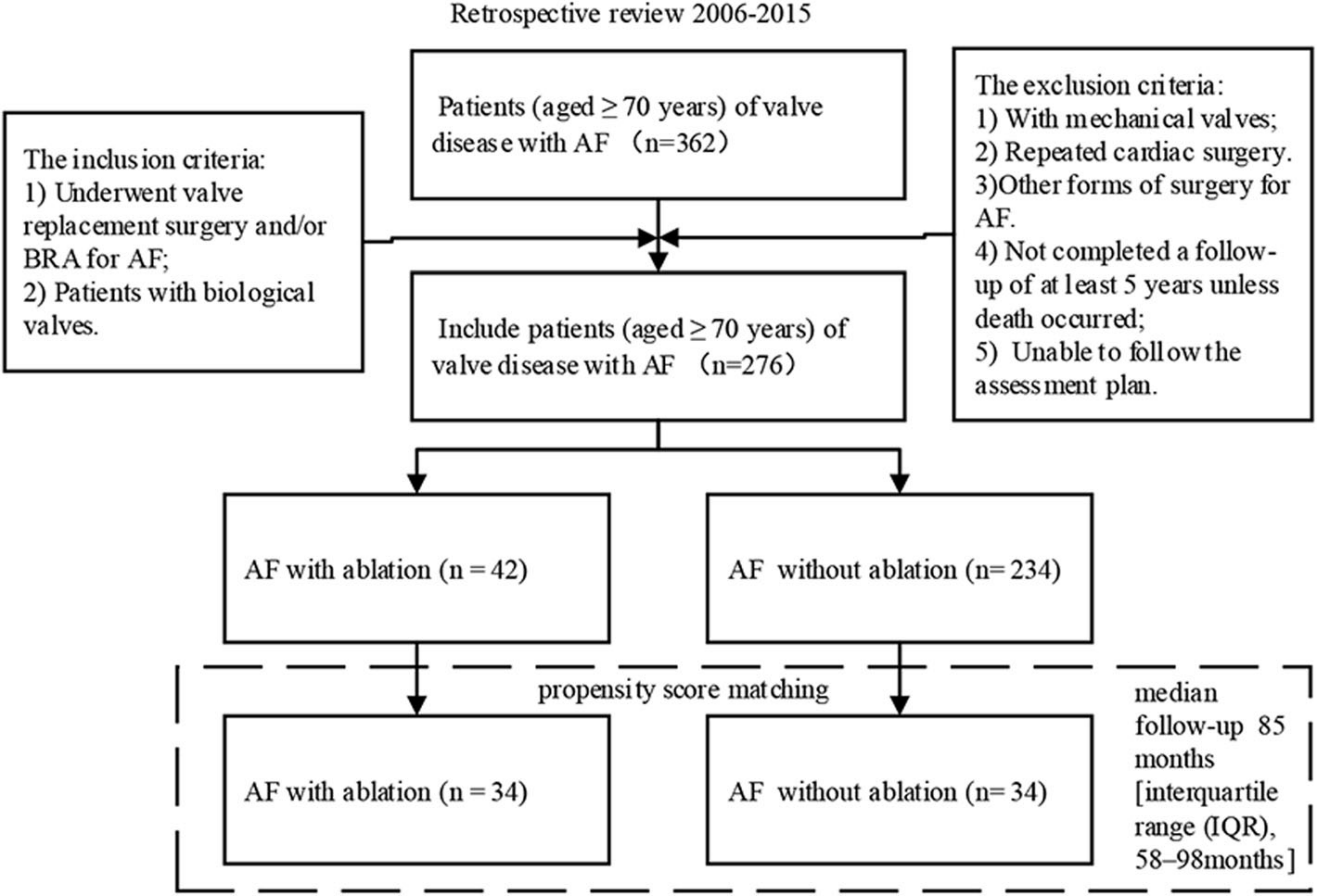
Flow diagram of the current study cohort

Between January 2006 and March 2015, a total of 362 consecutive patients (aged ≥70 years) with valve disease and AF underwent cardiac surgery at our institution. Among them, 48 patients underwent VR with concomitant Cox-maze procedure (42 underwent BRFA procedure and 6 underwent Cox-maze III procedure). The inclusion criteria were as follows: 1) Patients who underwent VR and/or BRFA for AF; and 2) patients with biological valves. The exclusion criteria were as follows: 1) Patients with mechanical valves; 2) patients who had received repeated cardiac surgeries; 3) patients who had received other forms of surgery for AF; and 4) patients who had not completed a follow-up of at least 5 years, unless death occurred. Finally, 276 patients were included in the final analyses. Preoperative demographic and clinical data, perioperative outcomes, and follow-up results were collected for all patients and retrospectively analysed. Of the 276 patients, 42 patients underwent VR with concomitant BRFA, and the remaining 234 patients underwent VR alone. All patients had persistent or long-lasting persistent AF, the diagnosis of which was confirmed by 24-h Holter monitoring before surgery. PSM was performed to achieve balance in covariates between groups. The process of patient selection is illustrated in Fig. 1.

The study was approved by the Ethics Committee of Union Hospital, Fujian Medical University, and the study adhered to the Declaration of Helsinki. The requirement for written informed consent from the patients or their guardians was waived due to the retrospective nature of the study.

### Surgical procedures

Decisions regarding the indication for AF ablation were made according to the preference of the heart surgeon at our institution (according to the patients’ EuroSCORE and other characteristics). In general, patients with a EuroSCORE ≥10, severe valvular heart disease requiring emergency surgery, LA diameter > 55 mm, or long-lasting (> 15 years) persistent AF were not considered suitable candidates for concomitant BRFA.

**Fig. 2:**
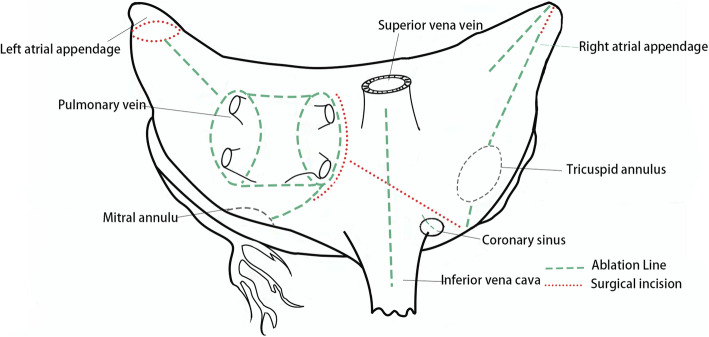
Lesion sets for the bipolar radiofrequency ablation procedure

All surgical procedures were performed through a median sternotomy under general anaesthesia with hypothermic cardiopulmonary bypass (CPB). We used the Medtronic Cardioblate G2 Surgical Ablation System (Medtronic Inc., Minneapolis, MN) with a bipolar radiofrequency clamp for surgical ablation in accordance with the method introduced by Sie et al. (Fig. 2) [18]. In cases where there was a thrombus in the left atrial, atrial thrombectomy was performed first after aortic cross-clamping. If there was no thrombus, right atrial ablation was performed with the heart beating, and left atrial ablation was performed during cardioplegic arrest. The right pulmonary was isolated, and the circumferential line around the right pulmonary veins was ablated. Two ablation lines were made from the right atrial appendage to the right atrial free wall and the tricuspid annulus (the junction between the anterior and septal leaflets of the tricuspid valve). The incision on the free wall of the right atrium was extended from the atrioventricular groove to the interatrial groove. Two ablation lines were created from the right atriotomy to the inferior vena cava and the superior vena cava. Another ablation line was created around the orifice of the coronary sinus. After aortic cross-clamping, the left pulmonary vein was isolated, and the ligament of Marshall was interrupted. The left atrium was entered via the incision parallel to the interatrial groove, and the circumferential line around the left pulmonary veins was ablated. Other ablation lines were created between the left atrial appendage and the left superior pulmonary vein, between the bilateral superior and inferior pulmonary veins, between the right inferior pulmonary vein and the posterior mitral annulus. The left auricle was finally ligated. Each radiofrequency ablation was performed at least three times. The valve prosthesis was then implanted. The temporary epicardial pacing wire was placed routinely after cardiac surgery.

### Postsurgical treatment

The postoperative care was similar for both groups. Unless contraindicated, amiodarone infusion was given 5 mg/kg in the first hour and 0.6 mg/kg/h over the following 72 h, and 200 mg orally twice a day for the next 3 weeks. For patients undergoing BRFA for AF, amiodarone infusion was given 200 mg orally daily until the end of the first 6 months to reduce the risk of early atrial arrhythmia recurrence. Sotalol or metoprolol was given as appropriate to patients with a history of side effects of amiodarone, or other clinical contraindications, in order to control the ventricular rate. Patients were advised to continue use of warfarin for 3 months after surgery, unless there was a specific contraindication. The duration of prothrombin was regularly monitored; the target international normalized ratio was 1.5–2.0 for aortic valve replacement and 2.0–2.5 for mitral or tricuspid valve replacement. In cases where the patient underwent valve replacement alone, and if no attempts were made to restore and maintain sinus rhythm by means of ablation, β-blocker drugs were administered to control the ventricular rate, and warfarin was used for anticoagulation.

### Outcome measures

The primary outcomes included all-cause death, cardiac death, sinus rhythm restoration, and stroke events. The secondary outcomes of interest were surgical mortality and major postoperative morbidity. Surgical mortality was defined as death during the same hospitalization, and cardiac death was defined as death due to malignant arrhythmia, stroke, congestive heart failure, or sudden unexplained death. Neurological complications are defined here as embolic or hemorrhagic strokes. Stroke was defined as an ischemic event caused by cerebral embolism. After 6 months of surgical ablation, any episode of AF, atrial flutter, or atrial tachycardia lasting more than 30 s recorded by electrocardiogram or 24-h Holter monitoring, was considered as recurrence of AF. Long-term survival was measured as time (months) to death from the date of surgery.

### Follow-up

Follow-up visits at discharge were made 3, 6, and 12 months postoperatively and annually thereafter. The patients’ health history, a physical examination, a 12-lead electrocardiogram, and an echocardiography were obtained at each visit. Patients with palpitations or documented atrial arrhythmias by 12-lead electrocardiography underwent 24-h Holter monitoring for further confirmation. The period between the day after surgery and death, loss to follow-up, or the predefined date of March 2020 was defined as the follow-up duration.

### Statistical analysis

Continuous variables are expressed as mean ± standard deviations and categorical variables are presented as count, unless otherwise noted. Differences were compared using the Student’s t-test or Mann-Whitney U test for continuous variables, and the chi-square test for categorical variables. One-to-one pair matching was performed using the caliper-matching method with the variables shown in Table 1**,** and a 0.02 propensity score tolerance was imposed on the maximum propensity score distance. The overall survival and AF-free survival were analysed using Kaplan–Meier analysis and log-rank methodology. Cox regression was used to test for differences in survival between groups, and hazard ratios (HR) and 95% confidence intervals (CI) were computed. The incidence of postoperative stroke in the matched population was estimated using the cumulative incidence function with death as a competing risk. The hazard of cardiac death was also analysed using the competing risk method, with non-cardiac death as the competing event. The differences between cumulative incidence curves in the competing risk analysis were compared using Gray’s tests. Fine and Gray competing risk regression was used to analyse the determinants of postoperative stroke and cardiac death. The sub distribution hazard ratio (SHR) and 95% CI were computed. A two-sided *P*-value < 0.05 was considered statistically significant. All statistical analyses were performed using SPSS 26.0 and R 3.6.2.

**Table 1:** Comparison of characteristics of AF ablated versus AF untreated populations: Total sample and propensity-matched groups

Variables		Total sample (*N* = 276)			Propensity-matched sample (*N* = 68)		
		AF with ablation (*N* = 42)	AF without ablation (*N* = 234)	P value	AF with ablation (N = 34)	AF without ablation (*N* = 34)	*P* value
Age (mean ± SD)		73.50 ± 2.44	74.89 ± 2.96	0.020	73.74 ± 2.50	74.15 ± 2.79	0.524
Male(n)		26	133	0.541	21	19	0.622
BMI		22.10 ± 3.13	21.96 ± 1.91	0.780	21.93 ± 3.01	21.43 ± 3.19	0.510
Smoking history(n)		15	49	0.037	11	12	0.798
Diabetes (n)		3	25	0.484	3	1	0.303
Hypertension (n)		23	87	0.032	16	15	0.808
LVEF		0.633 ± 0.860	0.635 ± 0.740	0.864	0.632 ± 0.088	0.654 ± 0.070	0.261
NYHA IV (n)		4	23	0.951			
LVDD (mm)		52.98 ± 4.62	54.78 ± 6.04	0.030	53.19 ± 4.44	53.34 ± 4.84	0.858
LA diameter (mm)		39.26 ± 9.13	39.39 ± 8.88	0.926	39.07 ± 8.88	38.86 ± 8.76	0.923
AF duration (years)		5.24 ± 2.75	9.95 ± 4.48	0.000	5.91 ± 2.58	5.18 ± 3.42	0.320
Serum creatinine (umol/L)		97.75 ± 22.07	96.69 ± 22.21	0.777	95.58 ± 22.80	94.22 ± 21.74	0.801
COPD(n)		2	4	0.212	1	0	0.314
Severe pulmonary hypertension (n)		7	47	0.607	7	3	0.171
Stroke/TIA(n)		8	23	0–081	7	4	0.323
CAD(n)		5	41	0.607	5	4	0.720
liver dysfunction (n)		3	3	0.016	1	0	0.314
Number of replacement valves (n)	Double	19	109	0.872	17	19	0.627
	Single	23	125		17	15	

*BMI* body mass index, *LVEF* left ventricular ejection fraction, *NYHA* New York Heart Association, *LVDD* left ventricular end diastolic dimension, *LAD* left atrial dimension, *COPD* chronic obstructive pulmonary disease, *TIA* Transient Ischemic Attack, *CAD* coronary artery disease

## Results

### Baseline characteristics

Patient characteristics and comparison of the data before and after matching are shown in Table 1. At our centre, approximately 13.3% of patients (aged ≥70 years) with valve disease with AF underwent concomitant Cox-maze surgery. PSM resulted in two groups of 68 patients (34 pairs), with similar baseline characteristics and a similar risk profile. The mean age of THE 34 pairs of patients was 73.94 ± 2.64 years. Before PSM, the duration of preoperative AF was significantly longer among patients with VR alone than among patients who underwent VR/BRFA. Moreover, age, smoking history, liver dysfunction, left ventricular diastolic diameter, and liver dysfunction were significantly different between the two groups.

### Operative data and early outcomes in the matched population

Table 2 shows the operative data and early outcomes of the study participants. The mean duration of CPB (108.94 ± 17.37 min vs 82.3 ± 17.47 min, P<0.001) and aortic cross-clamp (70.85 ± 14.93 min vs 48.71 ± 10.68 min, P<0.001) were significantly higher in the AF with ablation group. More patients in the AF with ablation group required prolonged mechanical ventilation. The AF with ablation group had a slightly longer duration of intensive care unit (ICU) stay (6.68 ± 4.48 days vs. 4.47 ± 4.56 days, *P* = 0.048) and a significantly longer length of hospital stay (17.76 ± 4.50 days vs. 13.44 ± 5.33 days, *P* = 0.001) compared to the AF without ablation group. There was no significant difference in hospital mortality between the two propensity-matched groups (5.88% vs. 2.94%, *P* = 0.555). Two patients died in the with ablation group (ventricular tachycardia in 1 case, low cardiac output syndrome and multiple organ failure in 1 case), and 1 patient died of pneumonia in the AF without ablation group. Other adverse events, such as pulmonary complications, neurological complications, renal failure requiring dialysis, low cardiac output, reoperation for bleeding, and permanent pacemaker (PPM) implantation were similar between the two matched groups. A PPM was implanted to treat third-degree atrioventricular block in three (7.14%) patients in the AF with ablation group within 6 months of surgery compared to one (2.38%) patient in the AF without ablation group (*P* = 0.303). The remaining eight patients who underwent concomitant BRFA and were not successfully matched experienced no severe perioperative complications or death, except for one patient who had a PPM implanted due to third-degree atrioventricular block. The incidence of PPM insertion in the AF with ablation group (*n* = 42, before PSM) was 9.52%.

**Table 2:** Operative Data and In-Hospital Outcomes in the matched population

Variables	AF with ablation (*N* = 34)	AF without ablation (*N* = 34)	*P* value
CPB time (min)	108.94 ± 17.37	82.3 ± 17.47	<0.001
Cross-clamp time (min)	70.85 ± 14.93	48.71 ± 10.68	<0.001
prolonged mechanical ventilation (> 72 h) (n)	15	7	0.038
Pulmonary complication (n)	29	26	0.355
Neurological complications^a^ (n)	5	2	0.231
Renal failure (n)	4	3	0.690
Dialysis (n)	2	1	0.555
ICU stay (days)	6.68 ± 4.48	4.47 ± 4.56	0.048
ICU readmission (n)	4	2	0.393
Length of hospital stay (days)	17.76 ± 4.50	13.44 ± 5.33	0.001
Low cardiac-output (n)	1	2	0.555
Reoperation for bleeding (n)	1	1	1.000
Hospitalized death cases (n)	2,	1	0.555

*CPB* cardiopulmonary bypass, *ICU* intensive care unit; ^a^ Neurological complications were defined here as embolic or hemorrhagic strokes

### Late outcomes in the matched population

Follow-up was complete after a median of 85.00 months (interquartile range [IQR], 58–98 months).

#### Long-term survival and cardiovascular death

**Fig. 3:**
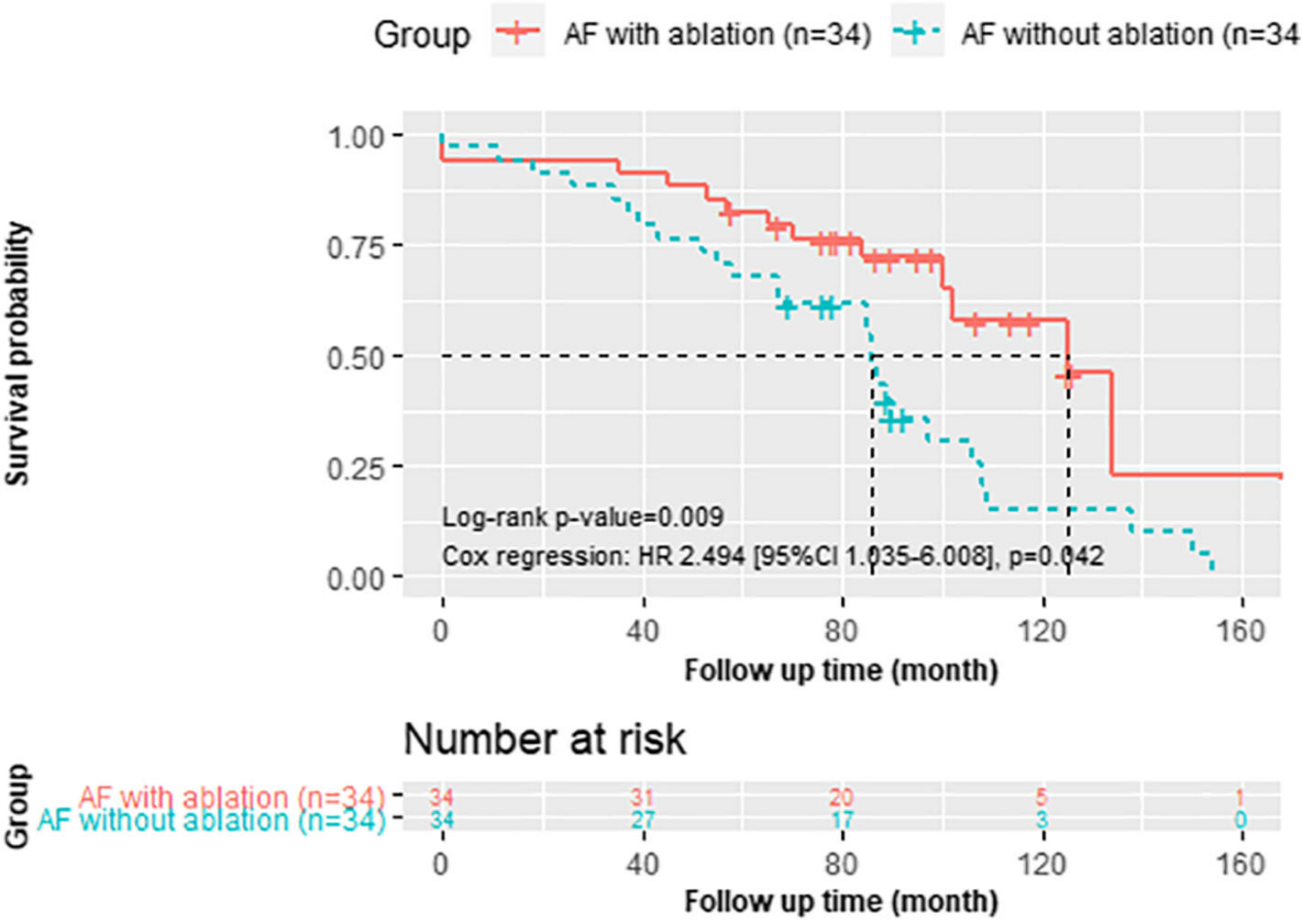
Survival of the matched AF with ablation group and AF without ablation group

**Fig. 4:**
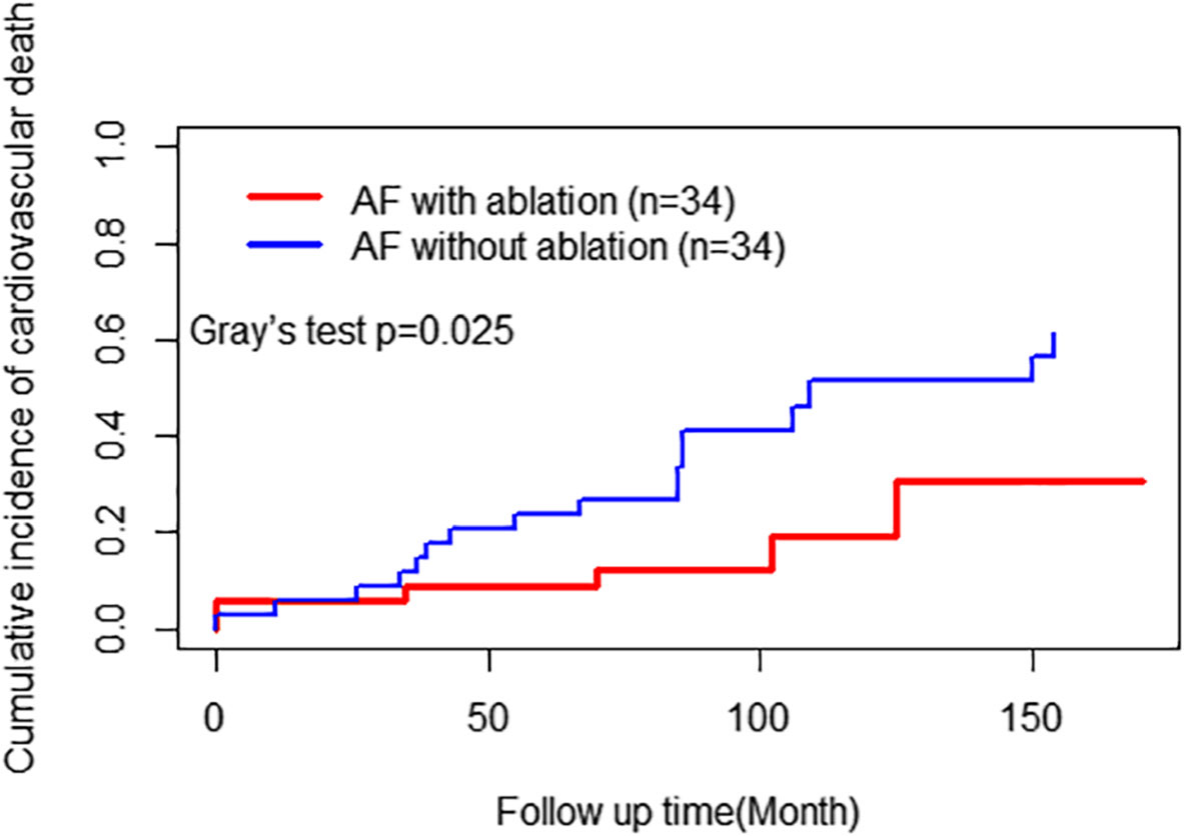
Cumulative incidence of cardiovascular death during follow up of the two propensity-matched groups

Kaplan–Meier analysis revealed significantly better overall survival (*P* = 0.009; Fig. 3) in the AF with ablation group during clinical follow-up (Cox regression: HR, 0.479; 95% CI, 0.235–0.974; *P* = 0.042). The 5- and 10-year survival rate was 82 and 58% for the AF ablation group compared to 68 and 15% for the AF without ablation group. Cardiovascular death occurred in 6 (17.6%) patients in the AF with ablation group, and 17 (50.0%) patients in the AF without ablation group. Cumulative incidence curves also showed a lower incidence of cardiovascular death in the AF with ablation group (*P* = 0.025, Gray’s test; Fig. 4). The unadjusted and adjusted SHRs evaluating the impact of additional BRFA procedures on the risk of cardiovascular death were 0.337 (95% CI, 0.135–0.843; *P* = 0.020) and 0.264 (95% CI, 0.093–0.753; *P* = 0.013), respectively.

#### Stroke events and sinus rhythm restoration

**Fig. 5:**
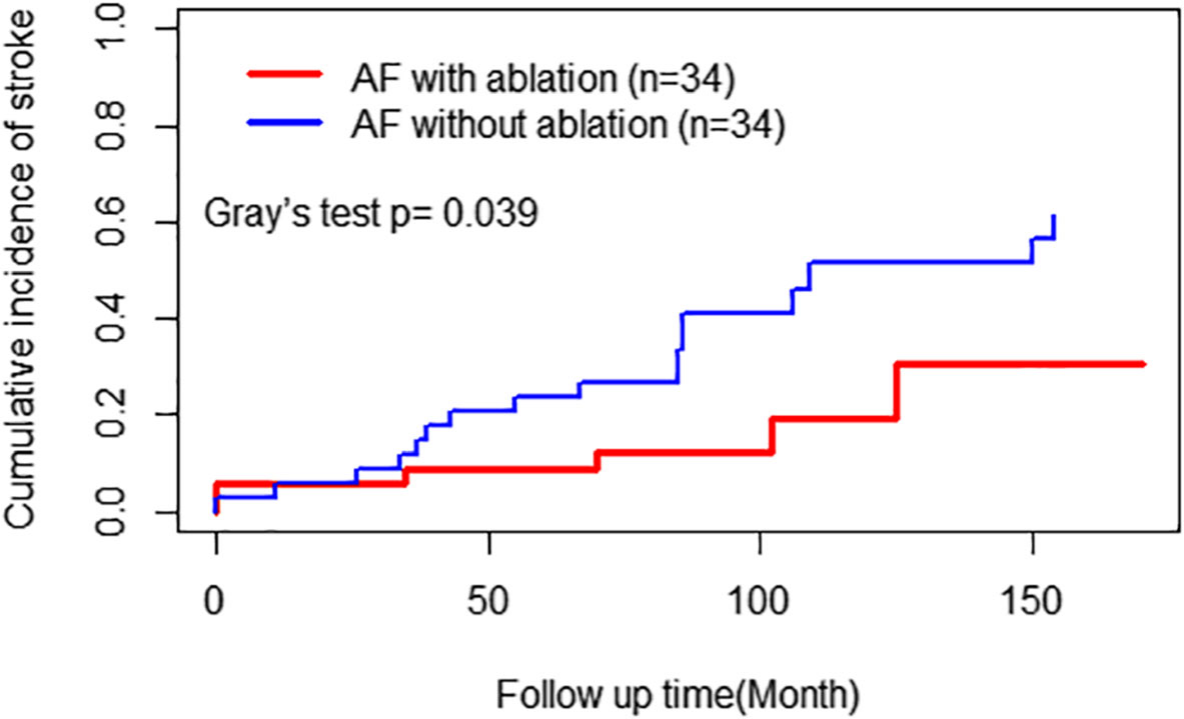
Cumulative incidence of stroke during follow up of the two propensity-matched groups

**Fig. 6:**
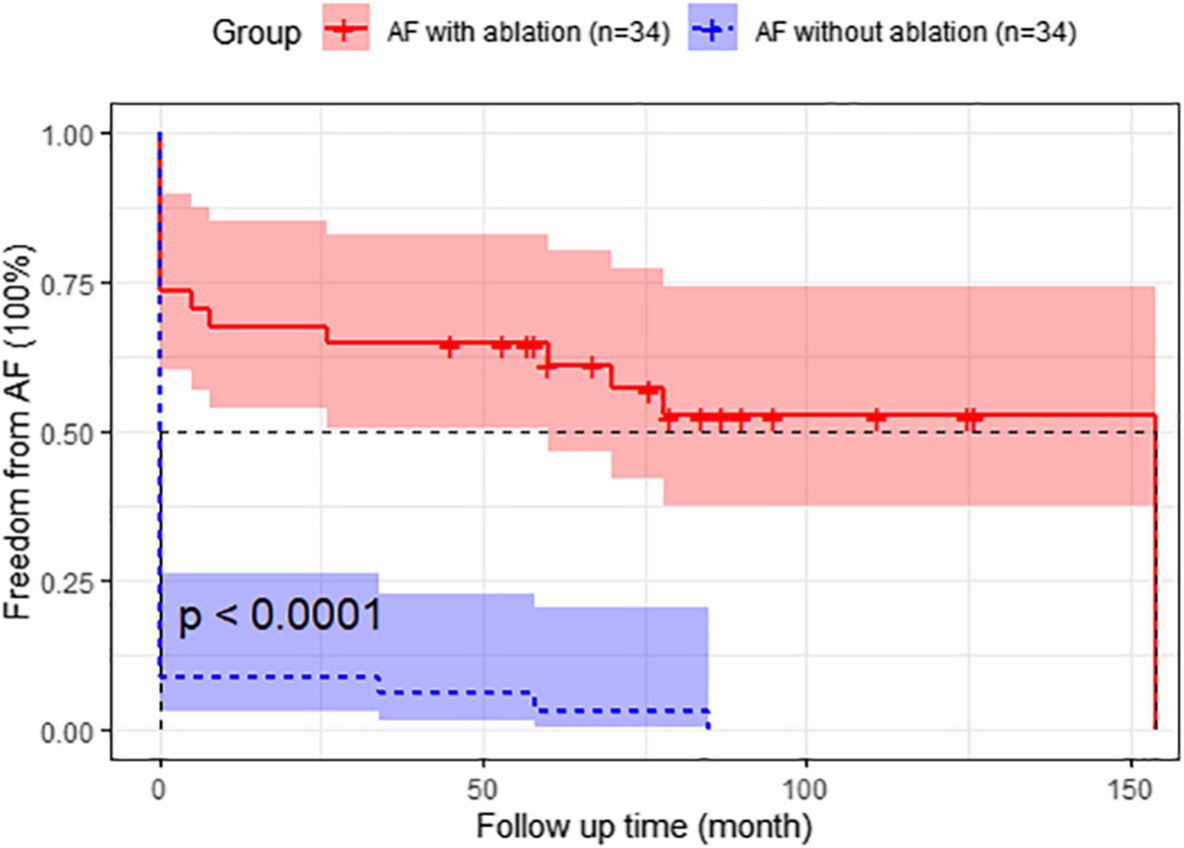
Freedom from AF during follow up of the two propensity-matched groups

Patients in the AF with ablation group had a reduced incidence of stroke events compared to patients in the AF without ablation group (*P* = 0.009, Gray’s test; Fig. 5) during clinical follow-up. Stroke events occurred in 5 (14.7%) patients in the AF with ablation group compared to 18 (52.9%) patients in the AF without ablation group. The unadjusted and adjusted SHRs evaluating the impact of additional BRFA procedures on the risk of stroke events were 0.343 (95% CI, 0.137–0.859; *P* = 0.022) and 0.219 (95% CI, 0.076–0.631; *P* = 0.005), respectively. The AF with ablation group had better AF-free survival than the AF without ablation group (P<0.0001; Fig. 6). Freedom from AF after 5 years was 58.0% in the AF with ablation group compared to 3.0% in the AF without ablation group, and some patients developed recurrent AF over time.

## Discussion

Valve disease with AF in the elderly will become more common as the elderly population progressively increases. However, surgical treatment of AF at the time of cardiac surgery is not yet widely practiced in elderly patients. Many surgeons believe that the surgical risks are increased, owing to the additional aortic cross-clamp and CPB time required for the addition of the Cox-maze procedure.

As a simpler surgical technique that only requires a few minutes of operative time, BRFA has been gradually used to create lines of conduction block in patients with AF who undergo other open-heart surgeries. Our study aimed to assess the efficacy, safety, and long-term survival of BRFA compared to VR without AF ablation in elderly patients (aged ≥70 years) with valve disease and AF.

The results of our matched control study indicate that satisfactory performance can be achieved by BRFA concomitant with VR, even in elderly patients, who are considered by many surgeons to be at high risk of ablation failure.

An important finding of our study is that the addition of BRFA procedures did not significantly increase the risk of common postoperative complications and postoperative mortality. One possible explanation is that better early postoperative hemodynamic stability associated with the treatment of AF. This result demonstrates that the addition of the BRFA procedure has the potential to eliminate the risks of increased CPB time. Our findings are in line with those previous studies. A study reported by Ad N and colleagues analysed the safety and efficacy of concomitant surgical AF ablation according to patients’ age, and showed that the performance of the Cox-maze III/IV procedure for patients aged ≥75 years did not increase the operative risk [19]. Another analysis of the Cox-maze procedure by Kuh JH and colleagues indicated that patients aged ≥70 years obtained satisfactory results without major morbidity or mortality, although this study lacked a control group for comparison [20]. In our study, the differences in clinical outcomes were illustrated by comparison of matched elderly patients who underwent valve replacement alone versus those who underwent an ablation concomitant valve replacement procedure. Furthermore, we observed some early clinical results that have not been reported in previous studies. We found that BRFA procedures for AF were correlated with an increased risk of prolonged mechanical ventilation after surgery. The length of hospital stay and ICU stay were also significantly longer. These findings may be related to impaired lung function due to the increased aortic cross-clamp and CPB time required for the addition of the BRFA procedure, which also prolongs the length of hospital stay and ICU stay. Another possible reason might be that patients in the AF with ablation group require a longer hospital stay to monitor the heart rhythm and heart rate.

The main indications for PPM implantation were a third-degree atrioventricular block, complications of which were due to ablation lines, which may damage the atrioventricular node [21]. Thus, biatrial ablation led to a significant PPM implantation rate. In our study, BRFA did not significantly increase the rate of PPI after surgery, which may be due to the fact that our surgeon was particularly careful to avoid damage of the atrioventricular node in the ablation area. The requirement for PPM has been reported to range from 4.5 to 13.3% in several studies [22–24]. In our cohort, 9.52% of patients received a PPM in the 6 months after surgery, which matched with the previous studies mentioned above. This suggests that relatively older age may not be a factor of the increased PPM requirements.

Another notable finding is that elderly patients had better long-term outcomes of RPFA concomitant with VR compared to VR without AF ablation. We show that the proportion of patients who achieved sinus rhythm was significantly higher in the AF with ablation group compared to the AF without ablation group, both at discharge and during follow-up. Moreover, the cumulative freedom from recurrence of AF was 65.2% at 5 years, which was comparable to that shown in previous studies conducted in experienced centres. Suwalski P et al. reported that the rate of sinus rhythm maintenance in 27 patients > 70 years old with a concurrent Cox-maze procedure was 89% at a mean follow-up of 51 months, [15] whereas our analysis resulted in a rate of 65.2%; this difference was likely driven by individual differences in patients and heterogeneity among ablation procedures. Furthermore, similar to our analysis, the investigators observed low in-hospital mortality. The study by Lee R. demonstrated that 78% of the patients (age 65.9 ± 12.2 years) who underwent surgical ablation were free of AF after 47 months of follow-up, and also proved that restoration of sinus rhythm improved survival [25]. In terms of long-term survival associated with concomitant ablation, the investigators reported a HR of 0.390, whereas the current study resulted in a very similar HR of 0.479. We also showed that additional BRFA procedures are associated with a low probability of stroke events and a significant decrease in cardiovascular death events and long-term overall mortality. We believe that restoration of sinus rhythm and excision of the left atrial appendage help to prevent stroke, which may lead to a remarkable reduction in long-term mortality. It has also been confirmed that chronic AF is associated with reduced survival after valve replacement [26]. The long-term success in our study argues strongly for a more widespread adoption of this currently underutilized treatment option for the elderly.

Previous clinical results of the concomitant BRFA with VR in the elderly were mostly compared to those in the younger group. Besides, previous studies have generally focused on patients with mitral valve surgery, and the outcomes of surgical ablation in patients with other types of valve surgery have not been well researched. What are the effects of different valve replacement types on clinical outcomes of bipolar radiofrequency ablation for atrial fibrillation? This is a question that deserves further exploration. The truth is that at present, this is beyond the scope of our study due to the limited number of elderly patients undergoing valvular and atrial fibrillation surgery in our center. Although several previous studies have described the efficacy and safety of concomitant surgical ablation with VR in elderly patients aged ≥70 years, to the best of our knowledge, our study is the first to compare matched elderly patients undergoing valve replacement alone versus an ablation concomitant valve replacement procedure. Our findings are robust because the duration of follow-up was adequate. Furthermore, we performed a matching-adjusted indirect comparison to remove observed differences in the patients’ baseline characteristics between the two groups. Indeed, PSM is being increasingly used to reduce possible confusion in observational studies [27], which can provide clinicians with relatively reliable comparative evidence to assist with decision-making. With no prospective randomized studies in the literature, this study might provide some reference value for clinical practice. We believe that the survival benefits of surgical radiofrequency ablation far outweigh the harmful effects of prolonged myocardial ischemic time and increased surgical complexity. Furthermore, the favourable in-hospital and long-term outcomes observed in our study add to the evidence that age alone should not be considered as a limiting factor for the concomitant BRFA procedure. However, further research is needed to confirm and validate the evidence.

There are several limitations to the current study. Firstly, this is a retrospective clinical analysis involving a small sample size at a single-centre. Due to these factors such as individual patient characteristics or surgeon’s preference/expertise, the majority of elderly patients do not accept ablation surgeries, and prefer conservative treatments for AF; for this reason, it was difficult to enrol a sufficient number of patients in this research. Moreover, we had to set strict inclusion criteria in order ensure the rigor of the research, which also reduced the number of suitable participants. Secondly, although we used PSM, confounds and selection biases between the two groups cannot be eliminated. Therefore, large-scale prospective randomized studies are warranted to evaluate BRFA in elderly patients. Further research is also needed to assess the quality of life of concomitant VR and BRFA in patients aged ≥70 years. Lastly, no comparison data for a younger population is presented in our study.

## Conclusion

In this study, we compared the outcomes between concomitant BRFA and no ablation in patients with valve replacement aged ≥70 years. Our results indicate that the addition of BRFA is both safe and feasible, even in elderly patients, with a better long-term survival and a reduced incidence of stroke events compared to valve replacement alone. These findings suggest that BRFA should always be considered as a concomitant procedure for AF in elderly patients who require to undergo cardiac surgery. However, a large-scale, prospective, multi-centre, randomized study should be performed in the future to fully validate our findings.

## Data Availability

All data generated or analyzed during this study are included in this published article.

## Abbreviations

AF: Atrial fibrillation
BRFA: Bipolar radiofrequency ablation
HR: Hazard ratios
SHR: Subdistribution hazard ratio
CI: Confidence intervals
VR: Valve replacement
CPB: Cardiopulmonary bypass
ICU: Intensive care unit
PPM: Permanent pacemaker
